# Association of Serum Creatinine Level with Prognosis of Laparotomy for Acute Mesenteric Ischemia after Cardiovascular Surgery

**DOI:** 10.1155/2022/1737161

**Published:** 2022-03-28

**Authors:** Yusuke Miyagawa, Yuta Yamamoto, Masato Kitazawa, Shigeo Tokumaru, Satoshi Nakamura, Makoto Koyama, Takehito Ehara, Nao Hondo, Yasuhiro Iijima, Yuji Soejima

**Affiliations:** Division of Gastroenterological, Hepato-Biliary-Pancreatic, Transplantation and Pediatric Surgery, Department of Surgery, Shinshu University School of Medicine, 3-1-1 Asahi, Matsumoto, Nagano 390-8621, Japan

## Abstract

**Introduction:**

Acute mesenteric ischemia is a life-threatening complication after cardiovascular surgery with a mortality rate of 52.9–81.3%. However, few studies have evaluated the predictors of clinical outcome after treatment for acute mesenteric ischemia following cardiovascular surgery. Therefore, this study aimed to elucidate prognostic factors in patients who underwent laparotomy for acute mesenteric ischemia after cardiovascular surgery.

**Methods:**

We retrospectively analyzed 29 patients (20 men and 9 women; median age, 71.0 years) who underwent laparotomy for acute mesenteric ischemia after cardiovascular surgery between January 2010 and August 2020. These patients were classified into the survivor group (comprising patients who were discharged or referred to another hospital, *n* = 16) and the nonsurvivor group (comprising those who experienced in-hospital mortality, *n* = 13). We compared clinical parameters between the groups to identify the predictors of outcomes.

**Results:**

More patients in the nonsurvivor group underwent emergency cardiovascular surgery (62.5% vs. 100%, *p* = 0.017) and received hemodialysis (12.5% vs. 61.5%, *p* = 0.008) at the onset of acute mesenteric ischemia than those in the survivor group. The prelaparotomy serum creatinine level was higher in the nonsurvivor group than in the survivor group (1.27 vs. 2.33 mg/dL, *p* = 0.004). Logistic regression analysis revealed an association between preoperative serum creatinine level and in-hospital mortality (odds ratio 5.047, *p* = 0.046), and Cox regression analysis demonstrated a relationship between serum creatinine level and in-hospital mortality (hazard ratio 1.610, *p* = 0.009). The area under the curve (receiver operating characteristic analysis) for the serum creatinine level was 0.813. Furthermore, the optimal cutoff value of the serum creatinine level was 1.59 mg/dL with a sensitivity and specificity of 0.846 and 0.687, respectively, in predicting in-hospital mortality.

**Conclusions:**

The elevated serum creatinine level was associated with a poor clinical outcome after surgery for acute mesenteric ischemia following cardiovascular surgery.

## 1. Introduction

Acute mesenteric ischemia (AMI) is characterized by sudden acute arterial or venous occlusion or a fall in circulating pressure, resulting in insufficient blood flow within the mesenteric circulation [1]. The mortality rate remains approximately 50%, despite improvements in multimodal treatment approaches, including endovascular techniques, over the past decade [2–4]. The estimated incidence of AMI was reportedly 1–3% after cardiovascular surgery (CS) [5–8]. Several factors, such as advanced age, hypertension, heart failure, prolonged ventilation, use of norepinephrine, and elevated serum levels of procalcitonin, myoglobin, lactate, and aspartate aminotransferase (AST), are reported to be risk factors for AMI after CS [9–11].

Renal failure [12] and a high Portsmouth physiological and operative severity score for the enumeration of mortality and morbidity (P-POSSUM) [13] indicate an elevated risk of mortality in AMI. In 1991, the POSSUM scoring system was established to predict postoperative complications and mortality using preoperative physiological scores and intraoperative surgical scores [14]. Furthermore, the mortality risk formula was modified to establish a P-POSSUM score that can predict a mortality rate more accurately [15]. The mortality rate in cases of AMI after CS was reportedly 52.9–81.3% [7, 9]. However, a few studies have evaluated predictors of clinical outcomes of patients after surgery for AMI following CS. Patients who underwent CS had various primary diseases, such as hypertension, heart failure, and diabetes mellitus. Therefore, we hypothesized that there are several predictive indicators of prognosis. The present study aimed to assess prognostic factors in patients who underwent surgery for AMI after CS.

## 2. Materials and Methods

### 2.1. Study Design

This retrospective cohort study included 29 patients who underwent laparotomy for AMI after CS at our hospital between January 2010 and August 2020. AMI was diagnosed based on clinical symptoms such as abdominal pain, ileus, and distension; laboratory test results indicating bowel necrosis; and triple-phase contrast-enhanced computed tomography (CT) demonstrating bowel ischemia. If contrast-enhanced CT was not performed, AMI was confirmed during surgery. Those who developed AMI during index CS were excluded. Regarding laparotomy for AMI, the primary surgery involved the resection of the ischemic intestine and ostomy using the remaining oral intestine. When the progression of necrosis or ischemia of the residual intestine was strongly suspected, a second surgery was performed.

Patients were classified into two groups as follows: the survivor group (*n* = 16), which comprised patients who were discharged or referred to another hospital, and the nonsurvivor group (*n* = 13), which comprised patients who experienced in-hospital mortality. We compared and examined clinical parameters between the two groups. Next, we conducted multiple logistic regression analysis and Cox proportional hazards regression analysis to determine prognostic indicators in patients who underwent laparotomy for AMI after CS. Finally, receiver operating characteristics (ROC) curves were generated to compare the prognostic indicators.

The P-POSSUM risk assessment method was used to calculate the rate of mortality after surgery for AMI following CS. The P-POSSUM scoring system comprises 12 physiology scores and 6 operative scores, and the formula for calculating the P-POSSUM-predicted mortality rate (*R*) [14, 15] is as follows:

(1) $$\begin{array}{c} \frac{\ln\;R}{1-R}=-9.065+0.1692\times PS+0.1550\times OS. \end{array}$$

### 2.2. Statistical Analysis

Statistical analysis was performed using the IBM® software Statistical Package for the Social Sciences, version 23.0 (IBM Corp., Armonk, NY, USA). Demographic data are presented as descriptive statistics. Comparisons between qualitative variables were conducted using the chi-square test and Fisher's exact test. Nonparametric data are presented as medians with interquartile ranges. The Mann–Whitney test was used to compare nonparametric data. Multiple logistic regression analysis was conducted to identify patient factors associated with in-hospital mortality using variables with a *p* value of <0.1 in univariate analysis. The multiple logistic regression analysis results are described as odds ratios with 95% confidence intervals (CIs). Additionally, Cox proportional hazards regression analysis was performed to evaluate the effect of several factors on survival after laparotomy for AMI following CS, using variables with a *p* value of <0.1 in the univariate analysis. The Cox proportional hazards regression analysis results are described as hazard ratios (HRs) with 95% CIs. A ROC curve was generated to investigate the discriminatory power of the equations in the analysis of mortality outcomes. The optimal cutoff value was considered the point closest to perfect differentiation (0, 1). After laparotomy for AMI following CS, survival rates were evaluated using the Kaplan–Meier product-limit method. All tests were two-tailed. Differences with a *p* value of <0.05 were considered statistically significant.

## 3. Results

During the study period, 2406 patients underwent elective or emergency CS. Among them, 29 patients (1.21%) underwent laparotomy for AMI after CS. The baseline characteristics of these patients are summarized in Table 1. The study cohort consisted of 20 male and 9 female patients with a median age of 71.0 years. The ratio of survivors to nonsurvivors was 16 : 13, and the in-hospital mortality rate was 44.8%. Regarding the details of the index CS, six patients (20.7%) underwent thoracic endovascular aneurysm repair, while five patients (17.2%) underwent abdominal endovascular aneurysm repair. Total arch replacement was performed for four patients (13.8%), while Y-graft replacement was performed for five patients (17.2%). Only one patient (3.5%) underwent cardiac surgery, which was coronary artery bypass grafting.

More patients in the nonsurvivor group underwent emergency CS (62.5% vs. 100%, *p* = 0.017) and received hemodialysis (12.5% vs. 61.5%, *p* = 0.008) at the AMI onset than those in the survivor group. Serum creatinine and AST levels prior to laparotomy for AMI were higher in the nonsurvivor group than in the survivor group (33.5 vs. 74.0 IU/L, *p* = 0.045, and 1.27 vs. 2.33 mg/dL, *p* = 0.004, respectively). No difference was observed in the proportion of patients with nonocclusive mesenteric ischemia (NOMI) (37.5% vs 46.2%, *p* = 0.638), as well as in P-POSSUM-predicted mortality rates (53.1% vs 97.7%, *p* = 0.092), between the two groups (Table 2).

The results of logistic regression analysis revealed that the serum creatinine level prior to laparotomy for AMI was significantly associated with in-hospital mortality after laparotomy (odds ratio 5.047, 95% CI 1.027–24.798, *p* = 0.046) (Table 3).

Cox regression analysis demonstrated that serum creatinine level and P-POSSUM-predicted mortality rate were associated with in-hospital mortality after laparotomy following CS (HR 1.610, 95% CI 1.124–2.308, *p* = 0.003 and HR 1.045, 95% CI 1.004–1.089, *v* = 0.033, respectively) (Table 4).

**Figure 1:**
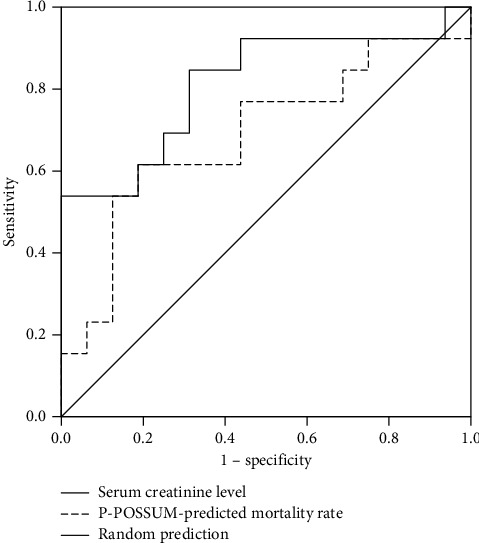
Receiver operating characteristic curves for the serum creatinine level (bold solid line) and the P-POSSUM-predicted mortality rate (bold dotted line). The area under the curve (AUC) of the serum creatinine level for in-hospital mortality is 0.813 (95% confidence interval [CI]: 0.646–0.979, *p* = 0.004) and that of P-POSSUM is 0.687 (95% CI: 0.483–0.892, *p* = 0.087). The optimal cutoff value of the serum creatinine level was 1.59 mg/dL with a sensitivity of 0.846 and a specificity of 0.687 to predict in-hospital mortality.

ROC analysis for the serum creatinine level showed an area under the curve (AUC) of 0.813 (95% CI: 0.646–0.979, *p* = 0.004), and ROC analysis for the P-POSSUM-predicted mortality rate demonstrated an AUC of 0.687 (95% CI: 0.483–0.892, *p* = 0.087) for in-hospital mortality. The optimal cutoff value of the serum creatinine level was 1.59 mg/dL with a sensitivity of 0.846 and a specificity of 0.687 to predict in-hospital mortality (Figure 1).

**Figure 2:**
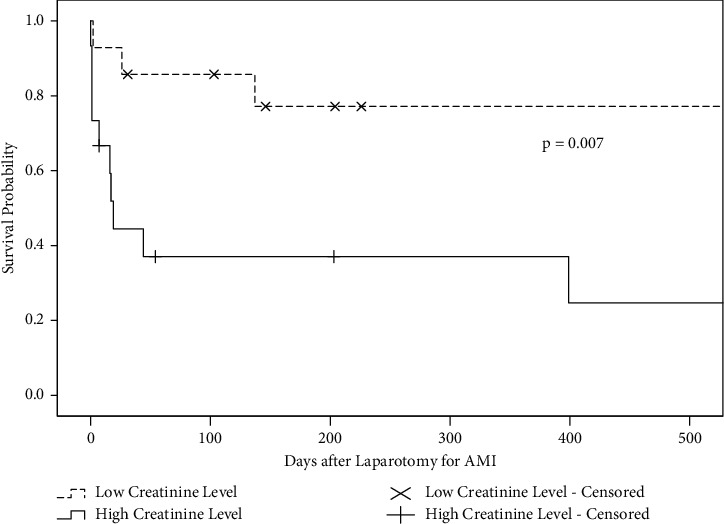
Survival probabilities in patients with a high creatinine level (≥1.59 mg/dL, *n* = 15) (solid line) and those with a low creatinine level (<1.59 mg/dL, *n* = 14) (dotted line) after laparotomy for acute mesenteric ischemia following cardiovascular surgery. The plus and cross marks represent censoring in patients with high and low creatinine levels, respectively.

The Kaplan–Meier estimator revealed that patients with a high creatinine level prior to laparotomy for AMI following CS (≥1.59 mg/dL, *n* = 15) had a shorter survival time after surgery for AMI than those with a low creatinine level (<1.59 mg/dL, *n* = 14) (*p* = 0.007) (Figure 2).

## 4. Discussion

This study demonstrated that a higher serum creatinine level was associated with poor clinical outcomes in patients who underwent laparotomy for AMI after CS. The serum creatinine level was superior to the P-POSSUM-predicted mortality rate in predicting in-hospital mortality. Our results appear clinically relevant because they indicate that, in a heterogeneous group of patients with AMI after CS with complex clinical courses, mortality can be predicted by assessing the serum creatinine levels prior to laparotomy for AMI.

Renal failure is reportedly associated with a high risk of AMI-related death postoperatively [12]. Furthermore, the serum level of fibroblast growth factor 23 (FGF-23) correlates with the occurrence and severity of NOMI after CS [16]. The serum level of FGF-23 is increased due to hyperphosphatemia in patients with renal failure and is associated with a high risk of mortality in patients with chronic kidney disease [17–20]. These findings suggest that an elevated serum creatinine level can be a reliable marker of the harmful effect of renal failure on the clinical outcome of AMI after CS.

POSSUM and P-POSSUM were designed to predict perioperative risk of general surgery [14, 15], and these scores are among the most common risk prediction models [21, 22]. As previously mentioned, the P-POSSUM scoring system has been reported to be useful for predicting the clinical outcome of patients with AMI [13]. Consistent with this result, in the present study, the P-POSSUM-predicted mortality rate was associated with mortality after laparotomy for AMI in Cox regression analysis.

AMI has two different etiological forms as follows: occlusive mesenteric ischemia (OMI), including arterial embolism, arterial thrombosis, and venous thrombosis, and NOMI [1]. NOMI is a disorder that causes ischemia and necrosis of the intestinal tract without organic obstruction in mesenteric blood vessels [23]; it is responsible for approximately 20% of the AMI cases [1]. The incidence of NOMI after CS is reportedly 0.6–9.0% [24–26], and the mortality rate accounts for 22.0–57.5% of all deaths [11, 25]. In the present study, the type of AMI (OMI or NOMI) did not affect the outcome of the study cohort. Although NOMI usually occurs in critically ill patients, it does not have a worse prognosis than OMI after CS.

The present study had some limitations. First, this was a single-center retrospective study with a small cohort, and this may have caused statistical errors. Second, only patients who underwent abdominal surgery for AMI were evaluated. Thus, the patient selection may have been biased. Third, the reason why the predictive value of the serum creatinine level for in-hospital mortality was superior to that of P-POSSUM in this cohort remains unclear. Therefore, further multicenter studies comprising a larger number of patients are needed to confirm the prognostic indicators of AMI after CS.

## 5. Conclusions

The present study demonstrated that a higher serum creatinine level prior to laparotomy for AMI after CS was associated with a poor clinical outcome. Furthermore, the serum creatinine level plays an important role in the prediction of in-hospital mortality after laparotomy for AMI following CS.

## Figures and Tables

**Table 1:** Patient characteristics.

Variables	Total (*n* = 29)
Sex	
Male (%)	20 (69.0)
Female (%)	9 (31.0)
Age, years^a^	71.0 (62.0–79.0)
Type of acute mesenteric ischemia	
Occlusive mesenteric ischemia, *n* (%)	17 (58.6)
Nonocclusive mesenteric ischemia, *n* (%)	12 (41.4)
Detailed procedure of index cardiovascular surgery	
CABG, *n* (%)	1 (3.5)
Total arch replacement, *n* (%)	4 (13.8)
Ascending aorta replacement, *n* (%)	1 (3.5)
Descending aorta replacement, *n* (%)	2 (6.9)
Y-graft replacement, *n* (%)	5 (17.2)
Thoracic endovascular aortic repair, *n* (%)	6 (20.7)
Endovascular aortic repair, *n* (%)	5 (17.2)
Treatment of peripheral artery, *n* (%)	5 (17.2)
Duration between cardiovascular surgery and acute mesenteric ischemia, days^a^	1.5 (0–41.3)
P-POSSUM-predicted mortality rate (%)^a^	82.0 (33.0–98.3)
Outcome after laparotomy	
Hospital discharge, *n* (%)	12 (41.4)
Hospital transfer, *n* (%)	4 (13.8)
In-hospital mortality, *n* (%)	13 (44.8)

^a^Median (interquartile range). CABG, coronary artery bypass grafting; P-POSSUM, Portsmouth physiological and operative severity score for the enumeration of mortality and morbidity.

**Table 2:** Comparison of the demographics of patients between the survivor and nonsurvivor groups.

Demographic characteristics	Survivor (*n* = 16)	Nonsurvivor (*n* = 13)	*p* value
Age, years^a^	78.0 (72.8–83.8)	77.0 (76.0–84.5)	0.779
Sex			
Male, *n* (%)	10 (62.5)	10 (76.9)	0.336
Female, *n* (%)	6 (37.5)	3 (23.1)	
Type of AMI			
Occlusive mesenteric ischemia, *n* (%)	10 (62.5)	7 (53.8)	0.638
Nonocclusive mesenteric ischemia, *n* (%)	6 (37.5)	6 (46.2)	
Duration between CS and AMI, days^a^	1.5 (0.0–47.25)	1.5 (1.0–37.00)	0.619
Operative type			
Cardiac surgery, *n* (%)	1 (6.3)	0 (0)	0.552
Thoracic aortic, *n* (%)	6 (37.5)	6 (46.2)	0.638
Abdominal aortic, *n* (%)	6 (37.5)	5 (38.5)	0.628
Peripheral artery, *n* (%)	3 (18.8)	2 (15.4)	0.604
Emergency CS			
Yes, *n* (%)	10 (62.5)	13 (100.0)	0.017
No, *n* (%)	6 (37.5)	0 (0)	
Comorbidities at the index CS			
Hypertension, *n* (%)	12 (75.0)	7 (53.8)	0.212
Diabetes mellitus, *n* (%)	4 (25.0)	2 (15.4)	0.435
Heart failure, *n* (%)	4 (25.0)	3 (23.1)	0.626
Peripheral artery disease, *n* (%)	3 (18.8)	1 (7.7)	0.383
Renal insufficiency, *n* (%)	2 (12.5)	3 (23.1)	0.396
Hemodialysis at the onset of AMI			
Yes, *n* (%)	2 (12.5)	8 (61.5)	0.008
No, *n* (%)	14 (87.5)	5 (38.5)	
Ventilator at the onset of AMI			
Yes, *n* (%)	5 (31.3)	5 (38.5)	0.493
No, *n* (%)	13 (68.7)	8 (61.5)	
Laboratory data prior to laparotomy for AMI			
White blood cell count,/*μ*L^a^	11085 (7908–14113)	10935 (6093–14898)	0.846
Lactate, mmol/L^a^	15.0 (10.5–63.0)	11.8 (9.6–84.8)	0.371
Creatinine, mg/dL^a^	1.27 (0.91–1.94)	2.23 (1.65–3.09)	0.004
AST, IU/L^a^	33.5 (20.5–85.3)	74.0 (41.0–273.3)	0.045
C-reactive protein, mg/dL^a^	8.07 (0.98–16.26)	9.47 (3.07–16.68)	0.619
CT findings at the AMI onset			
Ascites, *n* (%)	9 (56.3)	8 (61.5)	0.774
Free air, *n* (%)	2 (12.5)	2 (15.4)	0.617
Intestinal pneumatosis, *n* (%)	5 (31.3)	2 (15.4)	0.292
Hepatic portal vein gas, *n* (%)	1 (6.3)	1 (7.7)	0.704
P-POSSUM-predicted mortality rate^a^	53.1 (21.5–92.7)	97.7 (43.8–99.3)	0.092
Extent of bowel resection in laparotomy			
Small intestine, *n* (%)	7 (43.8)	9 (69.2)	0.170
Colorectum, *n* (%)	12 (75.0)	6 (46.2)	0.114

^a^Median (interquartile range). AMI, acute mesenteric ischemia; AST, aspartate aminotransferase; CS, cardiovascular surgery; CT, computed tomography; P-POSSUM, Portsmouth physiological and operative severity score for the enumeration of mortality and morbidity.

**Table 3:** Multiple logistic regression analysis of in-hospital mortality.

Variables	Univariate analysis			Multivariate analysis		
	Odds ratio	95% CI	*p* value	Odds ratio	95% CI	*p* value
Sex (male)	2.000	0.388–10.309	0.407			
Age	1.027	0.950–1.111	0.496			
Duration between CS and AMI	0.998	0.995–1.002	0.392			
Nonocclusive mesenteric ischemia	1.429	0.323–6.324	0.638			
Emergency cardiovascular surgery	2.1^*∗*^10^9^	—	0.999			
P-POSSUM-predicted mortality rate	1.015	0.993–1.038	0.184			
Hypertension	0.389	0.081–1.872	0.239			
Diabetes mellitus	0.545	0.083–3.590	0.528			
Heart failure	0.900	0.162–5.007	0.904			
ASO	0.361	0.033–3.962	0.405			
Renal failure	2.100	0.294–14.978	0.459			
Hemodialysis	11.200	1.751–71.637	0.011	6.353	0.745–54.195	0.091
Ventilator	1.375	0.295–6.402	0.685			
White blood cell count	1.000	1.000–1.000	0.669			
Lactate level	1.009	0.990–1.027	0.363			
Creatinine level	5.795	1.307–25.700	0.021	5.047	1.027–24.798	0.046
AST level	1.000	0.997–1.002	0.885			
CRP level	1.014	0.932–1.103	0.752			
Cardiac surgery	0.000	-	1.000			
Aortic surgery	1.833	0.279–12.066	0.528			
Peripheral artery	0.778	0.111–5.600	0.812			
Ascites	1.244	0.280–5.529	0.774			
Free air	1.273	0.154–10.530	0.823			
Intestinal emphysema	0.400	0.063–2.520	0.329			
Hepatic portal vein gas	1.250	0.071–22.132	0.879			
Resection of small intestine	2.893	0.622–13.455	0.176			
Resection of the colorectum	0.286	0.059–1.375	0.118			

AMI, acute mesenteric ischemia; ASO, arteriosclerosis obliterans; AST, aspartate aminotransferase; CRP, C-reactive protein; CS, cardiovascular surgery; CT, computed tomography; P-POSSUM, Portsmouth physiological and operative severity score for the enumeration of mortality and morbidity.

**Table 4:** Cox proportional hazard regression analysis of mortality.

Variables	Univariate analysis			Multivariate analysis		
	Hazard ratio	95% CI	*p* value	Hazard ratio	95% CI	*p* value
Sex (male)	1.808	0.388–10.309	0.369			
Age	1.021	0.966–1.080	0.456			
Duration between CS and AMI	0.999	0.996–1.002	0.435			
Nonocclusive mesenteric ischemia	1.292	0.432–3.858	0.647			
Emergency cardiovascular surgery	35.530	0.207–6085.317	0.174			
P-POSSUM-predicted mortality rate	1.016	0.997–1.034	0.094	1.045	1.004–1.089	0.033
Hypertension	0.535	0.179–1.603	0.264			
Diabetes mellitus	0.824	0.182–3.738	0.802			
Heart failure	0.796	0.218–2.904	0.730			
ASO	0.508	0.066–3.915	0.516			
Renal failure	1.642	0.448–6.019	0.454			
Hemodialysis	4.442	1.402–14.066	0.011	2.368	0.626–8.960	0.204
Ventilator	0.762	0.387–3.653	1.189			
White blood cell count	1.000	1.000–1.000	0.682			
Lactate level	1.008	0.996–1.021	0.176			
Creatinine level	1.538	1.158–2.042	0.003	1.610	1.124–2.308	0.009
AST level	1.000	0.998–1.001	0.820			
CRP level	1.003	0.945–1.065	0.916			
Cardiac surgery	0.046	0.000–7760.923	0.616			
Aortic surgery	0.908	0.198–4.173	0.901			
Peripheral artery	1.673	0.358–7.819	0.513			
Ascites	1.214	0.397–3.714	0.734			
Free air	1.661	0.362–7.628	0.514			
Intestinal emphysema	0.506	0.112–2.291	0.377			
Hepatic portal vein gas	0.902	0.116–7.021	0.921			
Resection of the small intestine	2.348	0.720–7.658	0.157			
Resection of the colorectum	0.466	0.156–1.392	0.172			

AMI, acute mesenteric ischemia; ASO, arteriosclerosis obliterans; AST, aspartate aminotransferase; CRP, C-reactive protein; CS, cardiovascular surgery; CT, computed tomography; P-POSSUM, Portsmouth physiological and operative severity score for the enumeration of mortality and morbidity.

## Data Availability

The data that support the findings of this study are available from the corresponding author upon reasonable request.
